# High Molecular Weight Proteins of *Trypanosoma cruzi* Reduce Cross-Reaction with *Leishmania* spp. in Serological Diagnosis Tests

**DOI:** 10.1155/2014/365403

**Published:** 2014-07-20

**Authors:** Alejandra Yunuen Cervantes-Landín, Ignacio Martínez, Muslim Schabib, Bertha Espinoza

**Affiliations:** ^1^Departamento de Inmunología, Instituto de Investigaciones Biomédicas, Universidad Nacional Autónoma de México C.U., Avenida Universidad No. 3000 Col., Deleg. Coyoacán, 04510 México, DF, Mexico; ^2^Centro Médico Nacional “La Raza”, Instituto Mexicano del Seguro Social, Calzada Vallejo Sin Número, Colonia La Raza, Deleg. Azcapotzalco, 02990 México, DF, Mexico

## Abstract

Chagas disease is caused by the parasite *Trypanosoma cruzi*. Because of its distribution throughout Latin America, sometimes it can overlap with other parasitic diseases, such as leishmaniasis, caused by *Leishmania* spp. This might represent a problem when performing serological diagnosis, because both parasites share antigens, resulting in cross-reactions. In the present work we evaluated Mexican sera samples: 83.8% of chagasic patients recognized at least one antigen of high molecular weight (>95 kDa) when evaluated by Western blot. Proteins of 130 kDa and 160 kDa are predominantly being recognized by asymptomatic chagasic patients. When the proteins were extracted using Triton X-100 detergent, a larger number of specific *T. cruzi* proteins were obtained. This protein fraction can be used to increase specificity to 100% in Western blot assays without losing sensitivity of the test. High molecular weight proteins of *T. cruzi* include glycoproteins with a great amount of *α*Man (*α*-mannose), *α*Glc (*α*-glucose), GlcNAc (N-acetylglucosamine), and *α*Gal (*α*-galactose) content and these structures play an essential role in antigens recognition by antibodies present in patients' sera.

## 1. Introduction

*Trypanosoma cruzi* is the causative agent of Chagas disease, endemic to many countries of Latin America and affecting millions of people. The parasite enters the body through broken skin and mucous membranes and causes acute but often mild symptoms. After a few months the chronic stage develops; however it can persist unnoticed for many years before causing abnormal heart rhythm, heart failure, digestive problems, and sudden cardiac death [1]. In this stage the detection of the parasite in peripheral blood is complicated, since* T. cruzi* is able to infect almost every cell of the host. Many serological tests are used for diagnosis, which detect specific antibodies against antigens of* T. cruzi*. In most of these techniques total extract of epimastigotes (insect stage of the parasite) is used as antigen with high levels of sensitivity, due to their easy culture which provides a large number of parasites, not like trypomastigotes' production which can be expensive and inefficient, increasing the cost of the diagnosis tests. But the use of epimastigotes protein extracts might cross-react with other infections, such as leishmaniasis caused by another trypanosomatid:* Leishmania* spp., so purified or recombinant antigens of different stages of* T. cruzi* have been tested [2] and still there is controversy about which antigen is the most efficient. In Mexico there are few reports about the geographic zones where Chagas disease and leishmaniasis may converge, but there have been some attempts to improve specificity of serological tests [3, 4].

The surface of this protozoan parasite is covered with a high density coat of glycoproteins, which contribute to both parasite protection and establishment of a persistent infection. Glycosylinositolphospholipids (GIPLs) and mucins represent the most abundant glycoconjugates in* T. cruzi* surface. Mucins are glycoproteins that bear a dense array of* O-*linked oligosaccharides which makes them well-suited for protection. During its life cycle,* T. cruzi* undergoes biochemical and morphological changes, including variation in the surface mucins and so in their biological activity. Other important glycoproteins present in* T. cruzi* surface are the trans-sialidases (TS), molecules able to transfer sialic acid residues from host glycoconjugates to parasite mucins [5].* Leishmania* spp. surface is covered with glycans as well, in order to survive the hostile environments to which it is exposed during its life cycle, being the most abundant the lipophosphoglycans in the promastigote stage [6]. Because of the antigenic similarities between both parasites, the aim of the present work was to find a protein fraction of a Mexican strain of* T. cruzi* that might reduce cross-reaction observed in serological diagnostic tests, without losing their sensitivity for routine diagnosis.

## 2. Materials and Methods

### 2.1. Parasite Culture and Antigen Preparation

Epimastigotes forms of* T. cruzi* (TBAR/MX/0000/Querétaro strain) were grown in liver infusion tryptose medium (LIT) supplemented with 10% of fetal bovine serum previously inactivated at 56°C for 30 min and 25 *μ*g/mL of hemine. Cultures were harvested at the log phase of growth [3].

Promastigotes of* Leishmania mexicana* were cultured in 199 medium supplemented with 10% of fetal bovine serum previously inactivated as described before, 1 M Hepes, 0.25% hemine, 50% triethanolamine, and 200 mM L-glutamine. Cultures were also harvested at the log phase of growth.

For the protein extracts preparation, parasites were collected by centrifugation at 2,000 g for 15 min at 4°C and divided into four fractions for different extraction methods. The parasites were washed and centrifuged twice in phosphate buffered saline (PBS) pH 7.2.

For proteins extraction by sonication, the pellet obtained after the last centrifugation cycle described before, was suspended in 5 mL of 10 mM Tris-HCl, pH 8.2 per gram of humid parasites with protease inhibitors (12 mM EDTA, 1 mM PMSF, 0.1 mM leupeptin, and 0.001 mM pepstatin). The parasites were sonicated three times for 1 min each. The mixture was centrifuged at 10,000 g for 30 min at 4°C. The supernatant was recovered and the protein concentration was determined using DC Protein Assay Kit (Bio-Rad Laboratories). The extract was stored at −20°C until use.

For Triton X-100 extraction, the pellet was suspended (2 × 10^6^ parasites/buffer *μ*L) in 1% Triton X-100 solution with protease inhibitors for 30 min at 4°C. The mixture was centrifuged at 10,000 g for 10 min at 4°C for supernatant recovering and the protein concentration was determined using DC Protein Assay Kit (Bio-Rad Laboratories). The extract was stored at −20°C until use.

For NP40 extraction, the pellet was suspended in 5 mL of 1% NP40 solution per gram of humid parasites with protease inhibitors. A vortex was used to shake the suspension for 15 seconds. The mixture was centrifuged at 10,000 g for 30 min at 4°C. The supernatant was then recovered and the protein concentration was determined using DC Protein Assay Kit (Bio-Rad Laboratories). The extract also was stored at −20°C until use.

For urea-thiourea extraction, the pellet was suspended in 3 mL of lysis buffer (7 M urea, 2 M thiourea, 4% CHAPS, and 120 *μ*M Tris) per gram of humid parasites with protease inhibitors. A vortex was used to shake the suspension for 3 min and then it was kept in ice for 10 min. The mixture was centrifuged at 10,000 g for 15 min and the supernatant was recovered. The protein concentration was determined using 2-D Quant kit. The extract was stored at −20°C until use.

### 2.2. Sera

**Figure 1:**
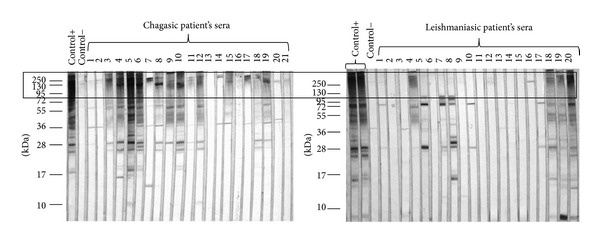
High molecular weight (>95 kDa) antigens predominantly recognized by chagasic patients antibodies. SDS-PAGE was performed in 12% polyacrilamide gel and then transferred into a nitrocellulose membrane for Western blot analysis of chagasic and leishmaniasic patients' sera (dilution 1 : 500) with sonicated total extract of epimastigotes of* T. cruzi* and peroxide-conjugated anti-human IgG (1 : 10000). Predominant recognition of antigens with molecular weights >95 kDa by chagasic patients' antibodies and less recognition by leishmaniasic patients' antibodies of the same antigens is highlighted (black box).

Sera used in this study come from 212 sera from Mexican infected patients who turned up from the* Centro Médico Nacional “La Raza”* of the* Instituto Mexicano del Seguro Social* with a positive result for Chagas disease by a commercially available ELISA test (Chagatest) for a confirmatory diagnosis to the* Laboratorio de estudios sobre Tripanosomiasis* at the* Instituto de Investigaciones Biomédicas*,* Universidad Nacional Autónoma de México* (UNAM). All of the blood donors were informed of the aim of the study and accepted to participate in it, signing an agreement. All sera were evaluated by ELISA and Western blot using sonicated total extract of epimastigotes of* T. cruzi*, according to the protocol described by Sánchez et al. [3]. Information of clinical features of patients used for experiments corresponding to Figures 1, 4, and 5 is shown in Table 1. A pool of well characterized positive patients' sera from Chagatest (Wiener lab; http://www.wiener-lab.com.ar/wiener/catalogo/archivos/6376_chagatest_elisa_recombinante_v3_0_en.pdf) was used as a positive control. As negative controls, sera of some laboratory members were used. Also a total of 27 sera samples of confirmed leishmaniasic patients were used.

### 2.3. Electrophoresis and Western Blot

Proteins of the four different* T. cruzi* extracts were separated by SDS-polyacrylamide gel electrophoresis (SDS-PAGE) and stained with Coomassie blue G250 or Silver staining. When required, Western blot assays were carried out by transferring the proteins in the polyacrylamide gel into nitrocellulose membranes. 12% acrylamide concentration was used when proteins of 10 kDa–250 kDa were visualized; 6% acrylamide concentration was used when proteins of high molecular weight (>95 kDa) were visualized. The membrane was cut in strips and left overnight in PBS containing 10% skimmed milk at 4°C with constant shaking. Individually, each strip was incubated with the serum to be evaluated, diluted 1 : 500 or 1 : 750 in PBS containing 10% skimmed milk for 2 h at room temperature. Each strip was washed three times with 0.1% Tween 20 in PBS solution and then incubated with peroxidase-conjugated anti-human IgG diluted 1 : 10000 at room temperature for 2 h. Three more washes were carried out and then the reaction was developed, adding 0.5 mg/mL of 3,3-diaminobenzidine in PBS and 0.02% H_2_O_2_. Distilled water was used in order to stop the reaction. Positive and negative control sera were included in each assay.

### 2.4. Lectin Blotting

After electrotransferring proteins into the nitrocellulose membranes, the strips were left for 2 h with 0.1% Tween 20 in PBS solution at room temperature and constant shaking. Each strip was then incubated with 5 *μ*g/mL of biotinilated lectins:* Concavalin A* (Con A),* Triticum vulgaris* (WGA),* Arachis hypogaea* (PNA),* Psophocarpus tetragonolobus* (PT),* Artocarpus integrifolia* (Jacalin), and* Maackia amurensis* (MAA) in 0.1% Tween 20 in PBS solution overnight at 4°C. Three washes were carried out as described in the Western blot method. Afterwards, each strip was incubated with peroxidase-conjugated streptavidin diluted 1 : 2000 in 0.1% Tween 20 in PBS for 2 h at room temperature. Three more washes were carried out and the reaction was developed as described before.

### 2.5. Enzymatic Deglycosylation

In order to remove all glycans of glycoconjugates present in the different protein extracts of* T. cruzi,* the Enzymatic Protein Deglycosylation kit (Sigma) was used. Briefly, 100 *μ*g of proteins was diluted in 30 *μ*L of miliQ water. 10 *μ*L of 5x reaction buffer was added, as well as 2.5 *μ*L of denaturation solution, then the mixure was gently mixed. Extracts were heated at 100°C for 5 min and cooled to room temperature, after which 2.5 *μ*L of the Triton X-100 solution was added. In order to remove sialic acid residues and* O-*glycans, 1 *μ*L of *α*(2 → 3,6, 8,9)-neuraminidase and 1 *μ*L of* O-*glycosidase were added. To remove galactose and N-acetylglucosamine residues, 1 *μ*L of both *β*(1 → 4)-galactosidase and *β*-N-acetylglucosaminidase was added. Each extract was incubated for 3 h at 37°C and then analyzed by electrophoresis or electrotransferred into a nitrocellulose membrane for Western blot assays.

### 2.6. Statistical Analysis

Student's *t*-test was carried out using SigmaStat 3.5 and GraphPad Prism 5 software. Significant levels were found at *P* < 0.001; *P* < 0.05.

## 3. Results

### 3.1. High Molecular Weight Antigens Are Recognized by Antibodies of Chagas Disease Patients

Sera samples were evaluated by ELISA and Western blot using as an antigen a sonicated total extract of* T. cruzi* epimastigotes to confirm and characterize them as positive to the infection. Only those samples with a positive result in both tests were considered for this study. In the Western blot evaluation, it was found that a major recognition of high molecular weight proteins by the antibodies is present in sera of chagasic patients: from 173 double positive sera samples evaluated, 83.8% (145) recognized at least one protein of high molecular weight (>95 kDa). Recognition in Western blot assays of these proteins by antibodies present in sera of leishmaniasic patients is considerably lower (Figure 1).

**Figure 2:**
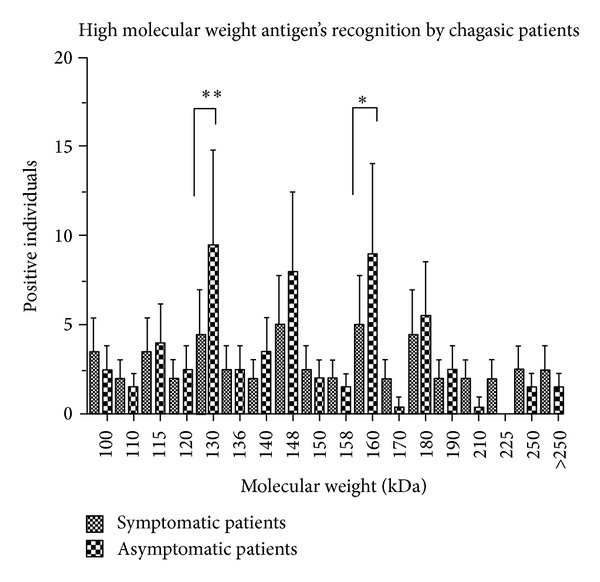
High molecular weight* T. cruzi* antigens' recognition by chagasic symptomatic and asymptomatic patients. Western blot analysis was performed on 173 chagasic patients' sera samples (1 : 500) with total sonicated extract of epimastigotes of* T. cruzi* and peroxide-conjugated anti-human IgG (1 : 10000). Molecular weight of each antigen recognized by symptomatic and asymptomatic patients was determined by comparison of molecular weight markers (Fermentas). Results represent media ± standard deviation. **P < 0.001; *P < 0.05.

Nevertheless, a heterogeneous recognition of high molecular weight proteins (>95 kDa) by antibodies of chagasic patients was observed. We found 18 different proteins recognized. The recognition of antigens by patients with a heart disease diagnosis and those who are asymptomatic was compared (Figure 2). There is a significant difference observed in the 130 kDa and 160 kDa antigens, which are being recognized preferentially by antibodies of asymptomatic patients. The 225 kDa antigen was recognized only by antibodies of 3 symptomatic patients.

### 3.2. High Molecular Weight Proteins Extracted by Different Methods

**Figure 3:**
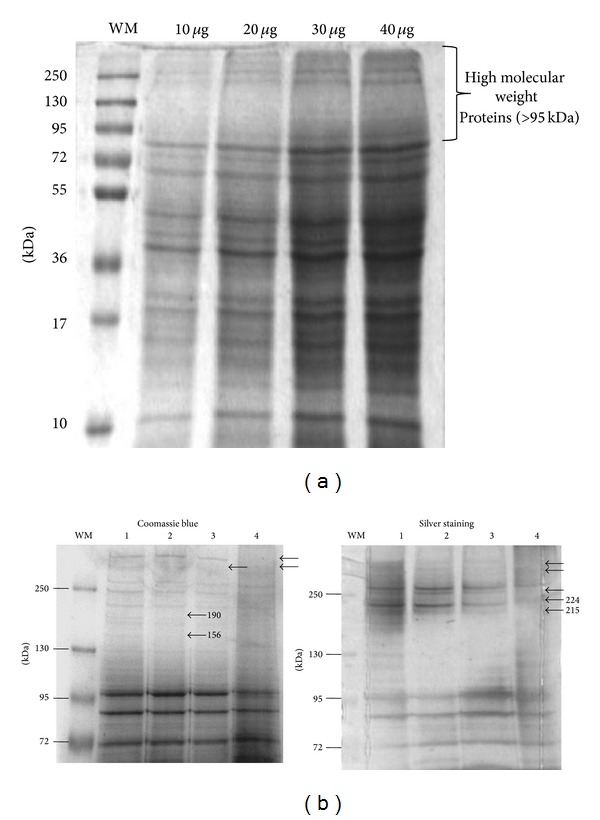
Electrophoresis of* T. cruzi* epimastigotes extracts. (a) SDS-PAGE of sonicated extract was performed in 12% polyacrylamide gel; each concentration has been pointed out. In all cases high molecular weight proteins (M.W. > 95 kDa) were found to be scarce. Gel was stained with Coomassie blue G250. (b) Electrophoresis of high molecular weight protein fractions of* T. cruzi* epimastigotes extracts. (1) Sonication. (2) Triton X-100 extraction. (3) NP40 extraction. (4) Urea-thiourea extraction. Concentration in each case was 15 *μ*g. SDS-PAGE was performed in 6% polyacrylamide gels. Differences between extracts and their molecular weight are highlighted (black arrows). Molecular weight markers (Fermentas) were used (WM), and gel was stained with Coomassie blue G250 or Silver staining.

High molecular weight proteins are recognized by most of the infected patients. But when the epimastigote sonicated total extract is separated by SDS-PAGE and stained with Coomassie Blue G250, few proteins with molecular weight >95 kDa are visualized (Figure 3(a)). Consequently, proteins were extracted using two detergents (Triton X-100, NP40) and two chaotropic agents (urea-thiourea) in order to try to obtain higher amounts of these proteins. Proteins were separated by SDS-PAGE in a 6% acrylamide concentration in order to improve high molecular weight proteins resolution (Figure 3(b)). Few differences were found between them. With Coomassie blue staining we observed a 190 kDa and a 156 kDa protein present only in the sonicated extract, as well as in the Triton X-100 extract. The Silver staining revealed a 224 kDa and a 215 kDa protein present in the sonicated, Triton X-100, and NP40 extracts but were absent in the urea-thiourea extract.

**Figure 4:**
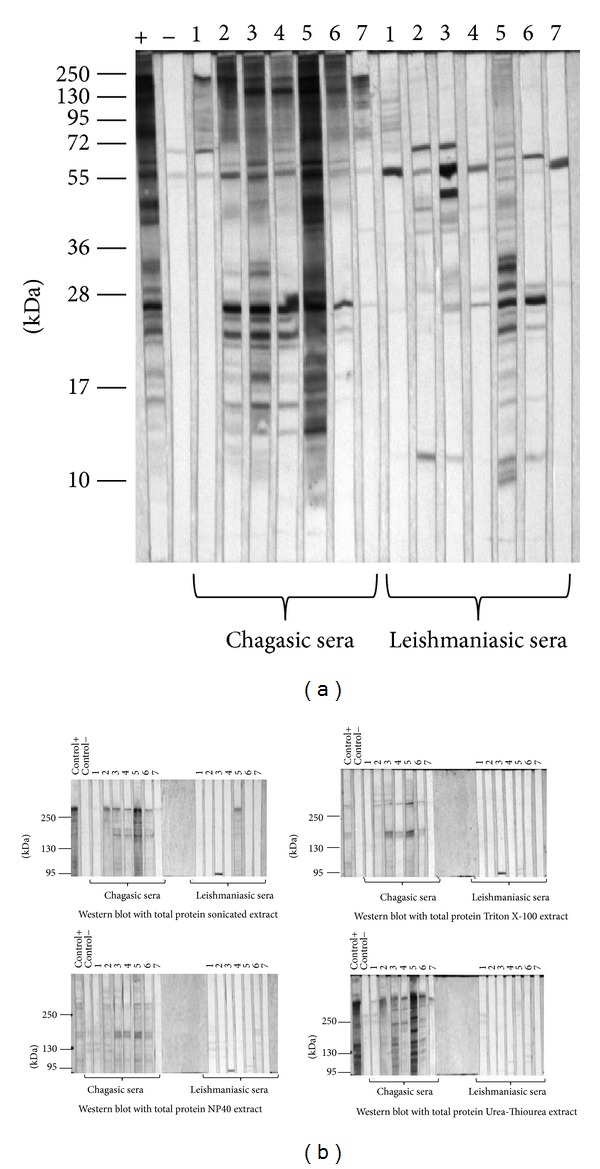
Western blot analysis of chagasic and leishmaniasic patients' sera using different extracts as antigens. (a) SDS-PAGE of sonicated extract was performed in 12% polyacrylamide gel and then transferred into a nitrocellulose membrane for Western blot analysis of the serum samples (dilution 1 : 500) and peroxide-conjugated anti-human IgG (1 : 10000). 100% of the leishmaniasic samples exhibit cross-reaction. (b) SDS-PAGE of sonicated, Triton X-100, NP40, and urea-thiourea extracts was performed in 6% polyacrilamide gel and then transferred into a nitrocellulose membrane for Western blot analysis of the serum samples (1 : 750) and the peroxide-conjugated anti-human IgG (1 : 10000). Unspecific proteins recognized by the negative control were not considered for the final analysis.

Then, 7 sera of chagasic patients and 7 sera of leishmaniasic patients were tested using total extract as well as each high molecular weight fraction. Proteins from each extract were separated using a 12% polyacrilamide gel in order to use the total extract and they were also separated in 6% polyacrilamide gels when it was required to maintain only high molecular weight proteins by SDS-PAGE. Then they were transferred into nitrocellulose membranes for Western blot assays. When using sonicated total extract, there were 5 out of 7 samples of leishmaniasic patients that cross-reacted (Figure 4(a)). Thus, we increased the sample dilution from 1 : 500 to 1 : 750 and used each high molecular weight fraction obtained by the different methods. Even though the number of recognized antigens was lower, all cases antibodies of chagasic patients recognized at least one antigen of high molecular weight in each fraction, maintaining the sensibility of the test. However, using these fractions the unspecific antigen recognition observed by the antibodies of leishmaniasic patients was reduced. High molecular weight proteins from Triton X-100 extraction display 100% sensibility and 100% specificity, since none of the leishmaniasic samples antibodies recognized any of the antigens of >95 kDa (Figure 4(b)).

### 3.3. Glycoconjugates Present in High Molecular Weight Proteins of* T. cruzi* and* Leishmania*

To determine the presence of glycoconjugates in the high molecular weight fractions of the different extracts, Lectin blot assays were developed. Molecular weights of those carbohydrates recognized by lectins that correspond to molecular weights of antigens recognized by antibodies of chagasic patients are shown in Table 2.

High molecular weight proteins of sonicated extract show an abundant glycan content; Con A and Jacalin bound several glycoproteins with *α*Man and *α*Gal, respectively. High molecular weight fraction of Triton X-100 and NP40 extraction turned out to be *α*Man rich as well. Few glycoproteins are extracted when using urea-thiourea buffer, since Con A and Jacalin bound only two carbohydrates each.

The glycan content in high molecular weight proteins of promastigotes of* Leishmania mexicana *sonicated extract was also determined. This protein fraction, analyzed by Lectin blot, showed a fewer number of glycoproteins. Con A lectin bound to *α*Man in the 190, 130, and 112 kDa molecular weights. PT lectin bound to carbohydrates in the 210, 200, and 112 kDa molecular weights.

### 3.4. Deglycosylation of Glycoproteins in the Extracts Diminishes the Antigen-Antibody Reaction in Western Blot

It was important to determine whether the carbohydrates of* T. cruzi* glycoproteins play an important role in antigenicity of the high molecular weight protein fractions. Deglycosylation was developed using an Enzymatic Protein Deglycosylation kit (Sigma) from the high molecular weight fraction of Triton X-100 extract, which previously showed to increase specificity when used as antigen in Western blot, and the fraction of the sonicated extract, which is commonly used as antigen in serological tests for diagnosis of Chagas disease.

**Figure 5:**
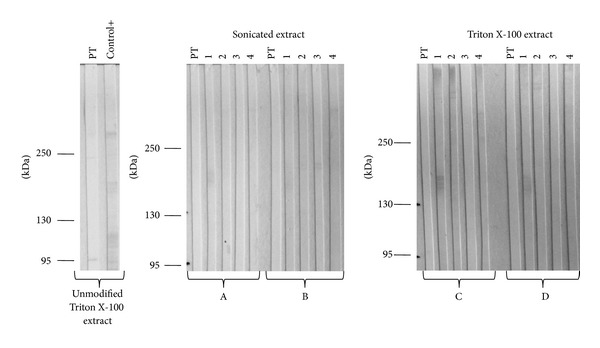
Western blot analysis of chagasic and leishmaniasic patients' sera using deglycosylated extracts. Enzymatic deglycosylation was performed with (A, C)* O-*glycosidase and *α*(2 → 3,6, 8,9)-neuraminidase; (B, D) *β*(1 → 4)-galactosidase and *β*-N-acetylglucosaminidase. 1 and 2 correspond to chagasic patients' sera samples and 3 and 4 correspond to leishmaniasic patients' sera samples (dilution 1 : 750) and peroxide-conjugated anti-human IgG (1 : 10000). Lectin PT was used as a positive control for carbohydrate recognition. A positive serum sample was also used as a positive control for antigenic recognition.

Sialic acid and* O-*glycans were removed from glycoproteins by adding *α*(2 → 3,6, 8,9)-neuraminidase and* O-*glycosidase. Galactose (gal) and N-acetylglucosamine (GlcNAc) residues were removed from the glycoproteins by adding *β*(1 → 4)-galactosidase and *β*-N-acetylglucosaminidase of both the Triton X-100 and the sonicated extract. Later, extracts were separated by SDS-PAGE in a 6% polyacrylamide gel. The deglycosylated proteins were transferred to a nitrocellulose membrane and incubated with chagasic and leishmaniasic sera samples. PT lectin was used as a positive control. Regardless of the treatment received, the antigens' recognition by the specific antibodies was almost completely lost in both cases (Figure 5).

## 4. Discussion

Trypanosomatids are the etiological agents of different infections and some of them share antigens recognized by antibodies present in patients' sera. Total extracts of epimastigotes are commonly used as antigens, since it is easier, less expensive, and more efficient to culture this parasite stage than trypomastigotes* in vitro*. Furthermore, since Chagas disease is considered to be a health problem mainly in rural locations of Latin American countries, where sometimes it is hard to count with the suitable equipment for diagnosis, efforts for developing simple, economic, and available serological tests must not be abandoned. It is well established that antibodies in patients sera are able to recognize* T. cruzi* antigens present in epimastigote stage as well [3].

Since high molecular weight proteins seem to be minor components in* T. cruzi* as observed by electrophoresis in the polyacrylamide gel, their purification might not be an easy task. For that reason, it should be considered to produce higher amounts of the antigens recognized by antibodies of Chagasic patients in a heterologous system.

In spite of the efforts for developing a sensible and specific test, high levels of false positive results are still obtained in some of serological diagnosis techniques [7–9]. Thus, it is important to keep looking for an antigen or group of antigens specific of* T. cruzi* that can improve specificity of the diagnostic tests.

When chagasic and leishmaniasic patients' sera were evaluated by Western blot, a predominant recognition of high molecular weight antigens by chagasic patients was found. About 83.8% of the patients recognized at least one antigen of molecular weight >95 kDa. Since each strain has specific characteristics and each patient develops antibodies against different antigens, a great heterogeneity was found when the molecular weight of the antigens recognized in this fraction was determined. Eighteen different proteins were found by Western blot and those of 130 kDa and 160 kDa are predominantly being recognized by asymptomatic patients' antibodies. It may be possible for these patients to possess a greater amount of antibodies against these antigens, so they have not developed any symptoms. Other studies must be carried out to establish if these antigens can be used as markers for protection of development of pathology or in order to produce a vaccine.

Proteins of high molecular weight from epimastigotes are not abundant when sonicated, so we tried to enrich the fraction with other extraction methods. Detergents' structure provides them with amphiphatic characteristics that allow them to aggregate in polar media forming micelles. In the cellular membrane, integral proteins and lipids are anchored on the lipid layer, but in detergent solutions, micelles' hydrophilic regions associate with proteins, extracting them from the membrane [10]. The ionic detergents, Triton X-100 and NP40, as well as the chaotropic agents, urea-thiourea, were used to produce three distinct protein extracts to be compared with the epimastigotes sonicated protein extract. Few differences were observed between them by SDS-PAGE. But when we used high molecular weight proteins fraction of each protein extract for Western blot analysis of chagasic and leishmaniasic patients, a reduced cross-reaction was found in most of the cases. Nevertheless high molecular weight proteins obtained by Triton X-100 extraction used as antigens in Western blot showed 100% sensitivity and increased specificity to 100%, abolishing false positives obtained when evaluating leishmaniasic patients' sera with sonicated total extract.

This fraction of high molecular weight proteins is predominantly being recognized by chagasic patients' antibodies. However many authors have described the great amount of glycoconjugates present in* T. cruzi* surface [11], which is covered with a rich coat of glycoconjugates, making them well-suited for protection and for the establishment of a persistent infection. Out of these glycoconjugates, mucins are the major components of the surface of* T. cruzi,* anchored to the outer phospholipid layer of the plasma membrane by glycosylphosphatidylinositol (GPI). Mucins have a threonine, serine, and proline rich sequence and their function changes according to the parasite stage. Trypomastigotes and amastigotes mucins are very similar; they have a protective role against proteases and participate in the adhesion and invasion of cells by trypomastigotes. tGPI-mucins (trypomastigotes-mucins) bear terminal Gal(*α*1,3)Gal epitopes that are a main target of antibody responses in chagasic patients. It has been found that, in the surface of epimastigotes of* T. cruzi* phylogenetic group I (such as Querétaro strain used in this study), it is possible to find the presence of galactofuranose. As humans do not produce glycoconjugates that contain this carbohydrate in particular, a strong immune response to it is induced [5].

Glycan content was determined in the high molecular weight region of the extracts. Lectins are carbohydrate binding proteins and can be used to discriminate and analyze the glycan structures of glycoproteins. The lectin blotting technique detects glycoproteins separated by SDS-PAGE and transferred to nitrocellulose membranes. Lectins of Con A, WGA, and Jacalin were those that bound to a greater number of glycan structures. In high molecular weight proteins of Qro strain, the most abundant carbohydrates were *α*Man, *α*Glc, GlcNAc, and *α*Gal. According to Atwood III et al., 2006,* T. cruzi* epimastigotes' high molecular weight proteins of organelle and plasma membrane/cytoplasmic fraction were also found to be *α*Man rich [12]. In other studies, it has been found that *α*Man is present as well in lower molecular weight (<90 kDa) antigenic proteins of epimastigotes of* T. cruzi* extracts [7].

When proteins were sonicated, a larger amount of glycoproteins whose molecular weight matches molecular weights of antigens recognized by patients' antibodies were obtained, in contrast to the other extraction methods performed in this study. Sonication process lyses cellular membrane in a mechanic way, cleaving the plasma membrane and releasing proteins. In this extract, the high molecular weight fraction contains predominantly *α*Man and *α*Gal glycoproteins. In a fewer amount, two glycoproteins with GlcNAc and NeuNAc were observed. In the same fraction, three glycoproteins with sialic acid residues of 250, 110, and  100 kDa were recognized by lectin of MAA. Triton X-100 extraction allowed obtaining glycoproteins with *α*Man, *α*Glc, GlcNAc, NeuNac, and sialic acid. In contrast, NP40 extraction showed only glycan binding to Con A and when extracting proteins with urea-thiourea, only two glycoproteins bound to Con A and Jacalin, respectively.

*L. mexicana* promastigotes sonicated extract was also analyzed by Lectin blot. There was found a fewer amount of glycoproteins bound to Con A. According to the molecular weight corresponding to the glycoproteins found, these might be present in the* T. cruzi* extract as well (190, 130, and 112 kDa). But glycoproteins of 210, 200, and 112 kDa with GalNAc and *β*Gal recognized by lectin of PT in* L. mexicana* extract were not present in the* T. cruzi* extract. The differences found in the carbohydrate content in high molecular weight proteins of both parasites might be crucial for the differences in the antigenicity of the fraction.

Since glycans can act as antigenic determinants, it was sought whether these structures are being recognized by chagasic and leishmaniasic patients' antibodies. Like Harth et al., 1992, it was found that some glycan structures of antigenic glycoproteins are essential for the antigen-antibody binding [13]. Using an enzymatic deglycosylation kit, glycans were removed from the glycoconjugates in the extract. Western blot analysis demonstrated that both chagasic and leishmaniasic antibodies failed to recognize antigens in the nitrocellulose membrane, so sensitivity of the test was lost. Interestingly, antigenicity may not be established by carbohydrates in other parasites' proteins.* Taenia solium* whole oncosphere antigens were deglycosylated* in situ* and used in Western blot assays. In that case, antigenic reactivity was not reduced [14].

## 5. Conclusions

In conclusion, high molecular weight proteins extracted with Triton X-100 turn out to be specific of* T. cruzi* when used as antigens in Western blot, since 100% sensitivity and specificity were accomplished. It is important to use the high molecular weight protein fraction in other serological techniques, such ELISA and DOT-ELISA used for diagnosis, and determine a possible increase in the specificity.

It is also essential to highlight the glycans participation as antigenic determinants. When carbohydrates are removed by enzymatic deglycosylation, the antigen recognition is lost and so the sensitivity of Western blot test. It will be interesting to study the immune response of Mexican patients to the glycan content of antigenic mixtures, since the trypanosomatid parasites are rich in this kind of molecules.

## Figures and Tables

**Table 1:** Mexican chagasic patients' sera information.

Sample number	Symptomatic	Western blot (protein bands)	ELISA test (OD/cut-off point)
1	symptomatic	3	1.45
2	US	>10	4.00
3	symptomatic	9	3.65
4	symptomatic	5	3.41
5	symptomatic	10	9.12
6	US	1	1.71
7	US	5	1.00

US: Unknown Status.

**Table 2:** Glycoproteins present in high molecular weight extracts fraction.

Extraction agent	Lectin	Recognized carbohydrate	Glycoprotein M.W. (kDa) recognized also by chagasic patients' antibodies
Sonication	Con A	*α*Man > *α*Glc > GlcNAc	>250, 190, 160, 130, 120, 110, and 100
	WGA	GlcNAc, NeuNAc	>250, 190
	Jacalin	*α*Gal→Ome	>250, 190, 130, 120, 110, and 100
	PT	GalNAc, *β*Gal	—
	PNA	*β*Gal (1→3) galNAc	—

Triton X-100	Con A	*α*Man > *α*Glc > GlcNAc	>250, 170, 160, 140, 120, and 110
	WGA	GlcNAc, NeuNAc	>250
	Jacalin	*α*Gal→Ome	—
	PT	GalNAc, *β*Gal	—
	PNA	*β*Gal (1→3) galNAc	—

NP40	Con A	*α*Man > *α*Glc > GlcNAc	>250, 170, 150, 120, 115, and 100
	WGA	GlcNAc, NeuNAc	—
	Jacalin	*α*Gal→Ome	—
	PT	GalNAc, *β*Gal	—
	PNA	*β*Gal (1→3) galNAc	—

Urea-thiourea	Con A	*α*Man > *α*Glc > GlcNAc	>250, 100
	WGA	GlcNAc, NeuNAc	—
	Jacalin	*α*Gal→Ome	130, 100
	PT	GalNAc, *β*Gal	—
	PNA	*β*Gal (1→3) galNAc	—

Lectin blot assays were performed to determine carbohydrate content in each fraction with a 5 *μ*g/mL concentration of lectins Con A, WGA, Jacalin, PT, PNA, and MAA and the peroxidase-conjugated streptavidin (dilution 1 : 2000).

## References

[1] da Silveira JF, Umezawa ES, Luquetti AO (2001). Chagas disease: recombinant *Trypanosoma cruzi* antigens for serological diagnosis. *Trends in Parasitology*.

[2] Clayton J (2010). Chagas disease 101. *Nature*.

[3] Sánchez B, Monteón V, Reyes PA, Espinoza B (2001). Standardization of Micro-Enzyme-Linked Immunosorbent Assay (ELISA) and Western blot for detection of *Trypanosoma cruzi* antibodies using extracts from Mexican strains as Antigens. *Archives of Medical Research*.

[4] Umezawa ES, Luquetti AO, Levitus G (2004). Serodiagnosis of chronic and acute chagas' disease with *Trypanosoma cruzi* recombinant proteins: results of a collaborative study in six Latin American Countries. *Journal of Clinical Microbiology*.

[5] Buscaglia CA, Campo VA, Frasch ACC, Di Noia JM (2006). *Trypanosoma cruzi* surface mucins: host-dependent coat diversity. *Nature Reviews*.

[6] McConville MJ, Mullin KA, Ilgoutz SC, Teasdale RD (2002). Secretory pathway of trypanosomatid parasites. *Microbiology and Molecular Biology Reviews*.

[7] Araujo FG (1986). Analysis of *Trypanosoma cruzi* antigens bound by specific antibodies and by antibodies to related trypanosomatids. *Infection and Immunity*.

[8] Lissaldo AM, Hoshino-Shimizu S, Umezawa ES, Stolf AM (1994). Alkaline soluble *Trypanosoma cruzi* epimastigote antigen (ASEA) applied to Dot-ELISA. *Revista do Instituto de Medicina Tropical de Sao Paulo*.

[9] Caballero ZC, Sousa OE, Marques WP, Saez-Alquezar A, Umezawa ES (2007). Evaluation of serological tests to identify *Trypanosoma cruzi* infection in humans and determine cross-reactivity with *Trypanosoma rangeli* and *Leishmania* spp.. *Clinical and Vaccine Immunology*.

[10] Caligur V (2008). Detergents and solubilization reagents. *Biofiles*.

[11] de Lederkremer RM, Agusti R (2009). Glycobiology of *Trypanosoma cruzi*. *Advances in Carbohydrate Chemistry and Biochemistry*.

[12] Atwood JA, Minning T, Ludolf F (2006). Glycoproteomics of Trypanosoma cruzi trypomastigotes using subcellular fractionation, lectin affinity, and stable isotope labeling. *Journal of Proteome Research*.

[13] Harth G, Mills AA, Souto-Padron T, De Souza W (1992). *Trypanosoma cruzi* glycoprotein 72: immunological analysis and cellular localization. *Molecular and Cellular Biochemistry*.

[14] Arana Y, Verastegui M, Tuero I, Grandjean L, Garcia HH, Gilman RH (2013). Characterization of the carbohydrate components of *Taenia solium* oncosphere proteins and their role in the antigenicity. *Parasitology Research*.

